# Analysis of Sheng-Mai-San, a Ginseng-Containing Multiple Components Traditional Chinese Herbal Medicine Using Liquid Chromatography Tandem Mass Spectrometry and Physical Examination by Electron and Light Microscopies

**DOI:** 10.3390/molecules21091159

**Published:** 2016-09-01

**Authors:** Yung-Yi Cheng, Tung-Hu Tsai

**Affiliations:** 1 Institute of Traditional Medicine, National Yang-Ming University, Taipei 112, Taiwan; vininecheng@gmail.com; 2 Graduate Institute of Acupuncture Science, China Medical University, Taichung 404, Taiwan; 3 School of Pharmacy, College of Pharmacy, Kaohsiung Medical University, Kaohsiung 807, Taiwan; 4 Department of Chemical Engineering, National United University, Miaoli 36063, Taiwan; 5 Department of Education and Research, Taipei City Hospital, Taipei 103, Taiwan

**Keywords:** Sheng-Mai-San, mass spectrometry, ginsenoside, pharmaceutical herbal products

## Abstract

Sheng-Mai-San is a multi-component traditional Chinese herbal preparation. Due to the fact granulated additives, such as starch, carboxymethyl cellulose, lactose and raw herbal powder may alter the content of the bioactive markers in the herbal products, a developed ultra-high performance liquid chromatography tandem mass spectrometry (UHPLC-MS/MS) method was used to measure the herbal biomarkers of ginsenoside Rb_1_, Rb_2_, Rc, Rd, Re, Rg_1_, Rh_1_, compound K, ophiopogonin D and schizandrin from the Sheng-Mai-San herbal formulation. Besides, scanning electron microscopy (SEM) was used to observe the morphology of the herbal granular powders. Light microscopy with Congo red and iodine-KI reagent staining was used to identify the cellulose fiber and cornstarch added to pharmaceutical herbal products. The swelling power (SP), water solubility index (WSI), and crude fiber analysis were used to determine the contents of cellulose fiber and cornstarch in pharmaceutical herbal products. In this study, we developed a novel skill to assess the quantification of appended cornstarch in pharmaceutical herbal products using Aperio ImageScope software. Compared with the traditional cornstarch analysis, our analysis method is a rapid, simple and conversion process which could be applied to detect the percentage of added cornstarch in unknown powder products. The various range of the herbal content for the five pharmaceutical manufacturers varied by up to several hundreds-fold. The physical examination reveals that the morphology of the herbal pharmaceutical products is rough and irregular with sharp layers. This study provides a reference standard operating procedure guide for the quality control of the Chinese herbal pharmaceutical products of Sheng-Mai-San.

## 1. Introduction

Traditional Chinese herbal medicine is one of the most important forms of complementary and alternative medicine (CAM) used for health care in the world. Sheng-Mai-San is a traditional Chinese herbal prescription, originally invented by the ancient Chinese physician Gao Li (also known as Dongyuan Li; 1180–1251). Recent studies have shown its effectiveness in the treatment of heat-stroke and cardiovascular diseases. Sheng-Mai-San contains three herbs: *Panax ginseng* (red ginseng), *Fructus schisandrae* (Chinese herbal name: wu-wei-zi) and *Radix ophiopogonis* (Chinese herbal name: mai-men-dong). Recently, Shenmai injection, a botanical pharmaceutical product comprising an alcoholic extract of *Panax ginseng* and *Ophiopogon japonicus*, has been used for the treatment of chronic pulmonary heart disease [1,2,3,4]. Due to its injection formulation, the quality control of herbal pharmaceutical products in terms of safety and activity is an important concern. HPLC/MS/MS has been applied for the detection of herbal ingredients in biological samples [5,6,7]. Ginseng is a popular nutrition supplement and well-known traditional herbal medicine. Ginseng herbal extracts have been used as tonic, sedative, anti-fatigue, and anti-gastric ulcer drugs for thousands of years in Asia [8]. The herbal ingredients of ginsenosides have been considered as the main effective components responsible for their antidiabetic, anti-inflammatory, and antitumor activities [7,8,9,10,11]. Ginsenosides Rb_1_, Rb_2_, Rc, Rd, Re, Rf, and Rg_1_ are the major bioactive constituents of ginseng. *Fructus schisandrae* extract has been used as treatment agents for hepatotoxicity, asthma, cancer, and diabetes mellitus [12,13,14]. Schizandrin is one of the major effective compounds of *Fructus schisandrae*. Previous reports have indicated that schizandrin has many biological properties, including neuroprotective, hepatoprotective, anti-inflammatory, antitumor, and antioxidation activities [14,15,16]. According to The Divine Farmer’s Materia Medica, an ancient Chinese herbal medicine book, *Ophiopogon japonicus* is sweet and balanced. It has been used for the treatment of acute and chronic inflammation and cardiovascular diseases for thousands of years [17]. Ophiopogonin D, a major bioactive saponin of *Ophiopogon japonicus* is regarded as a biomarker for quantification of this herb.

High-performance liquid chromatography (HPLC) is suitable technique for the quality control of herbal medicine. Ultra-performance liquid chromatography with diode array detection (UHPLC-DAD) [18], and tandem mass spectrometry (LC-MS/MS) [6,7,8,19,20] have been applied to analyze the herbal ingredients in Sheng-Mai-San extract. To examine the quality of Chinese herbal preparations in the form of herbal extracts and industrial pharmaceutical products, a validated chemical and physical analysis method is required. A survey from the PubMed database using quality control of herbal medicine as a keyword yielded 1657 articles. Meanwhile, quality control of Sheng-Mai-San yielded eight articles. However, no article was found using the keyword chemical and physical analysis of Sheng-Mai-San.

The aim of this study was to develop a standard procedure to ensure the quality of the multiple components of the Chinese herbal medicine Sheng-Mai-San. The validated LC-MS/MS method was applied to determine the contents of herbal ingredients and compare the quality of commercially available herbal industrial pharmaceutical products from various pharmaceutical manufacturers. Moreover, physical examination was applied to inspect the product quality of Sheng-Mai-San. In addition to evaluating the physical quality characteristics of each brand of industrial pharmaceutical herbal products, the observation of outer appearance, the degree of swelling, solubility, and crude fiber contents were examined by the following methods: (1) scanning electron microscopy (SEM); (2) light microscopy photographs with Congo red staining; (3) light microscopy photographs with iodine-KI staining; (4) water solubility index (WSI); (5) swelling power (SP); and (6) crude fiber analysis.

## 2. Results and Discussion

### 2.1. Optimization of LC-MS/MS Conditions

**Figure 1:**
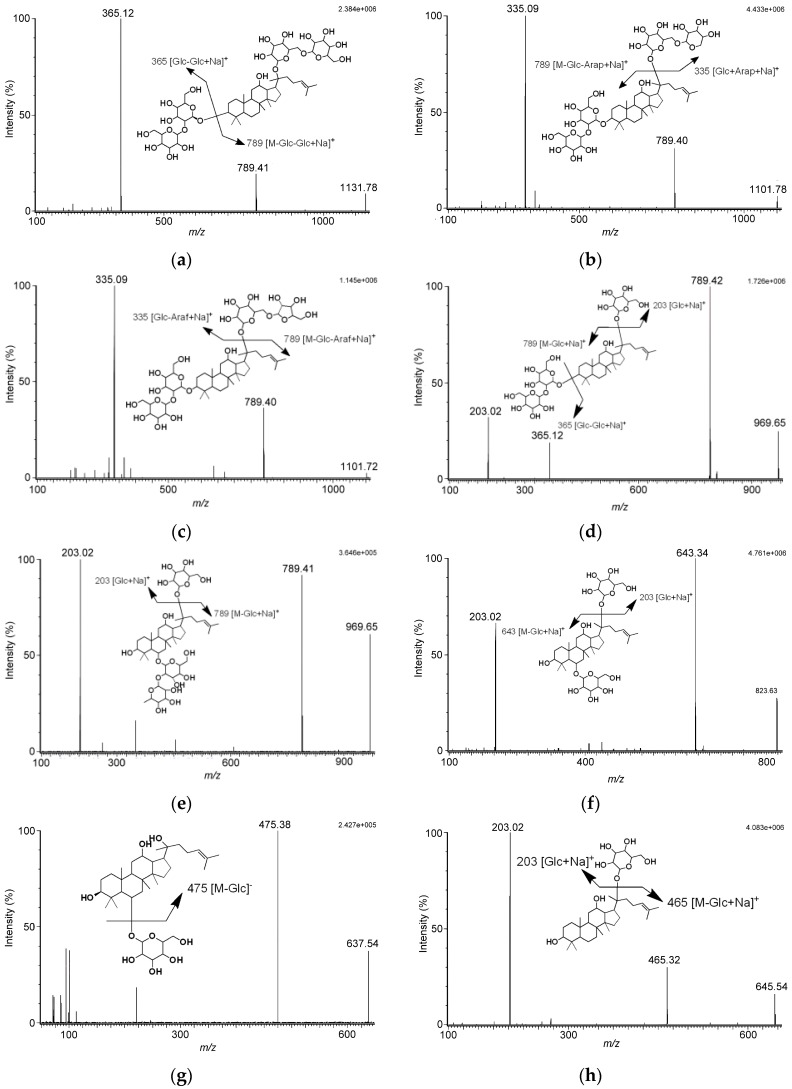

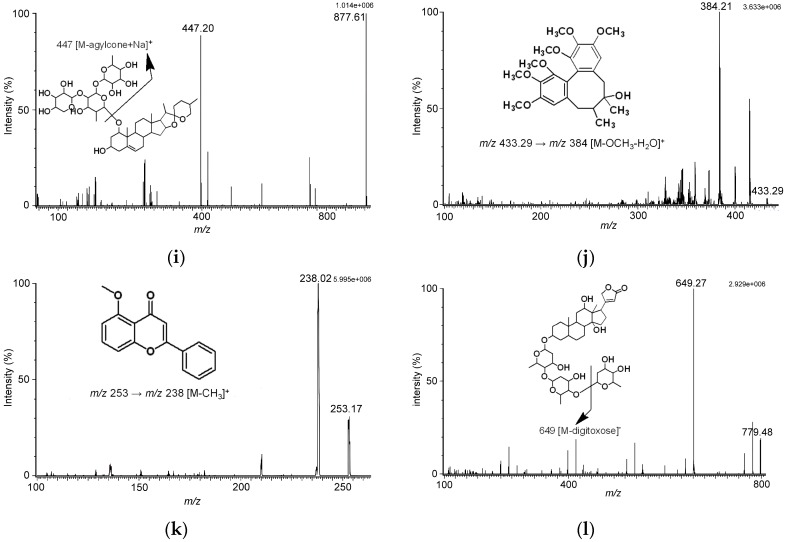
Product ion mass spectra of ten marker compounds: (**a**) ginsenoside Rb_1_; (**b**) ginsenoside Rb_2_; (**c**) ginsenoside Rc; (**d**) ginsenoside Rd; (**e**) ginsenoside Re; (**f**) ginsenoside Rg_1_; (**g**) ginsenoside Rh_1_; (**h**) ginsenoside compound K; (**i**) ophopogonin D; (**j**) schizandrin; and the internal standards (**k**) 5-methoxyflavone and (**l**) digoxin.

To identify and quantify the Sheng-Mai-San marker ingredients, a ultra-high performance liquid chromatography tandem mass spectrometry (UHPLC-MS/MS) method was developed. The electrospray ionization (ESI-MS) spectra of the experimental and marker compounds were acquired in both ESI(+) and ESI(−) ionization modes simultaneously. Analytes were quantified in selective reaction monitoring (SRM) mode: ginsenoside Rb_1_, *m*/*z* 1131.78 [M + Na]^+^ → *m*/*z* 789.41 (CE 56 eV); ginsenoside Rb_2_, 1101.78 [M + Na]^+^ → *m*/*z* 789.40 (collision energy (CE) 54 eV); ginsenoside Rc, 1101.78 [M + Na]^+^ → *m*/*z* 789.46 (CE 58 eV); ginsenoside Rd, 969.65 [M + Na]^+^ → *m*/*z* 789.40 (CE 54 eV); ginsenoside Re, 969.65 [M + Na]^+^ → *m*/*z* 789.41 (CE 44 eV); ginsenoside Rg_1_, 823.63 [M + Na]^+^ → *m*/*z* 643.34 (CE 36 eV); ginsenoside Rh_1_, 637.48 [M − H]^−^ → *m*/*z* 475.32 (CE 24 eV); ginsenoside compound K, 645.54 [M + Na]^+^ → *m*/*z* 465.32 (CE 30 eV); ophiopogonin D, 877.61 [M + Na]^+^ → *m*/*z* 447.20 (CE 50 eV); schizandrin, 433.29 [M + H]^+^ → *m*/*z* 384.21 (CE 20 eV); 5-methoxyflavone, 253.17 [M + H]^+^ → *m*/*z* 238.02 (CE 24 eV); digoxin 779.48 [M − H]^−^ → *m*/*z* 649.27 (CE 34 eV) (Table 1; Figure 1). Analyzing the tandem mass spectrometry (MS/MS) spectra of analytes revealed fragmentation pattern of ginsenosides lost monosaccharide or disaccharide units, that were the characteristic fragments for the triterpene type of ginsenosides. As the MS/MS spectra in Figure 1 show, we found no matter whether the MS conditions were set in positive or negative ion mode, the cleavage pathway of ginsenosides occurred at the glycosidic bond.

The fragmentation patterns of ginsenosides obtained in our study were the same as the fragmentation patterns of ginsenosides reported by Perrault and Costello [21,22]. The fragmentation pattern of ophiopogonin D indicated the precursor ion of *m*/*z* 877, [M + Na]^+^, lost the agylcone to yield the product ion of *m*/*z* 447, [M − agylcone + Na]^+^. Besides, the fragmentation pattern of schizandrin suggested the loss of a methyl group and a water molecule occurred, giving the product ion of *m*/*z* 384, [M − OCH_3_ − H_2_O]^+^.

**Figure 2:**
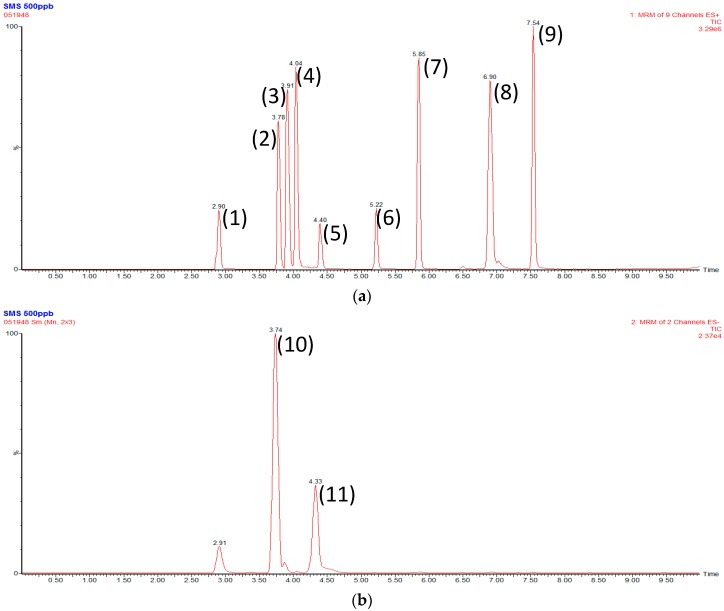
Selective reaction monitoring (SRM) chromatograms of the analytes. (**a**) Positive mode: (1) Ginsenoside Re and Rg_1_, (2) Ginsenoside Rb_1_, (3) Ginsenoside Rc, (4) Ginsenoside Rb_2_, (5) Ginsenoside Rd, (6) 5-methoxyflavone, (7) Schizandrin, (8) Ophiopogonin D, (9) Ginsenoside Compound K; (**b**) Negative mode: (10) Digoxin, (11) Ginsenoside Rh_1_.

The analyte separation was optimized by varying the gradient elution, proper column, and flow rate, which were considered to be pivotal factors. The chromatographic conditions provided good resolution, appropriate ionization, and coelution. Optimization of LC-MS/MS conditions is required to provide good sensitivity, selectivity, and peak symmetry (Figure 2).

### 2.2. Method Validation

The calibration curves showed good linearity in the ranges of 25–500 ng/mL for ginsenoside Rb_1_, 50–500 ng/mL for ginsenoside Rb_2_, 50–500 ng/mL for ginsenoside Rc, 10–500 ng/mL for ginsenoside Rd, 25–250 ng/mL for ginsenoside Re, 10–100 ng/mL for ginsenoside Rg_1_, 50–500 ng/mL for ginsenoside Rh_1_, 25–250 ng/mL for ginsenoside compound K, 10–100 ng/mL for ophiopogonin D, and 50–500 ng/mL for schizandrin. The calibration curves and correlation coefficients (*r*^2^) were as follows: *y* = 0.0059*x* − 0.047 (*r*^2^ = 0.999, ginsenoside Rb_1_), *y* = 0.0156*x* + 0.6078 (*r*^2^ = 0.998, ginsenoside Rb_2_), *y* = 0.0235*x* − 0.9627 (*r*^2^ = 0.998, ginsenoside Rc), *y* = 0.0108*x* − 0.0355 (*r*^2^ = 0.999, ginsenoside Rd), *y* = 0.011*x* − 0.0143 (*r*^2^ = 0.998, ginsenoside Re), *y* = 0.0018*x* − 0.0121 (*r*^2^ = 0.999, ginsenoside Rg_1_), *y* = 0.002*x* − 0.0305 (*r*^2^ = 0.999, ginsenoside Rh_1_), *y* = 0.0285*x* + 0.0203 (*r*^2^ = 0.999, ginsenoside compound K), *y* = 0.0309*x* + 0.0775 (*r*^2^ = 0.998, ophiopogonin D), and *y* = 0.0126*x* − 0.2304 (*r*^2^ = 0.999, schizandrin) (Table 2).

The limit of detection (LOD) for the herbal ingredients is shown in Table 2. The data show that th limit of quantification (LOQ) for ginsenoside Rb_1_, ginsenoside Rb_2_, ginsenoside Rc, ginsenoside Rd, ginsenoside Re, ginsenoside Rg_1_, ginsenoside Rh_1_, ginsenoside compound K, ophiopogonin D, and schizandrin were 25, 50, 50, 10, 25, 10, 50, 25, 10 and 50 ng/mL, respectively. The precision and accuracy were evaluated by intra- and inter-day assays. Good linearity was achieved over the calibration range, with all coefficients of correlation being greater than 0.995. The relative standard deviation (RSD) values were found to be within the range of 0.45%–8.37% for intraday assays and 0.09%–12.06% for inter-day assays, with accuracy ranges of 4.44%–3.41% and 7.22%–11.01%, respectively. The results are summarized in Table 3, which indicates that the precision and accuracy values were within the acceptable range.

### 2.3. Determination of the Markers for Sheng-Mai-San Preparations

To investigate the contents of the marker components in commercially available Sheng-Mai-San products from various pharmaceutical manufacturers, the most strongly detected product ion was selected for quantification analysis. The results demonstrate that, among the samples obtained from pharmaceutical manufacturers A–E and the decoction powder, the contents of ginsenoside Rb_1_, ginsenoside Rb_2_, ginsenoside Rc, ginsenoside Rd, ginsenoside Re, ginsenoside Rg_1_, ginsenoside Rh_1_, ophiopogonin D, and schizandrin were 9.44 to 346 μg/g, 6.57 to 277.6 μg/g, 6.53 to 544.8 μg/g, 19.58 to 244.8 μg/g, 26.46 to 738.7 μg/g, 4.60 to 114.6 μg/g, 33.38 to 550.6 μg/g, 2.65 to 16.36 μg/g, and 7.39 to 1532 μg/g, respectively (Table 4). However, the ginsenoside compound K herbal ingredient was not detectable in any of the samples from the pharmaceutical manufacturers or the decoction powder. The ophiopogonin D herbal ingredient was only detectable in the samples from pharmaceutical manufacturers A, D and E. All established contents for the potential active ingredients in the samples are shown in Table 5. LC-MS/MS with chemical profiling was conducted to rapidly evaluate the chemical consistency among Sheng-Mai-San herbal pharmaceutical products (SMS products). The composition ratios of this herbal pharmaceutical product from different manufacturers were consistently labeled, but samples A–E contained different amounts of the ten marker ingredients. The herbal origin, herbal growth environment, period of cultivation, decoction process, and even granulation process influenced the contents of the marker components. In this study, a developed and validated LC-MS/MS method was used to simultaneously determine ginsenoside Rb_1_, ginsenoside Rb_2_, ginsenoside Rc, ginsenoside Rd, ginsenoside Re, ginsenoside Rg_1_, ginsenoside Rh_1_, ginsenoside compound K, ophiopogonin D, and schizandrin in various brands of Sheng-Mai-San.

### 2.4. Evaluation of Additives for Raw Herbal Powder

**Figure 3:**
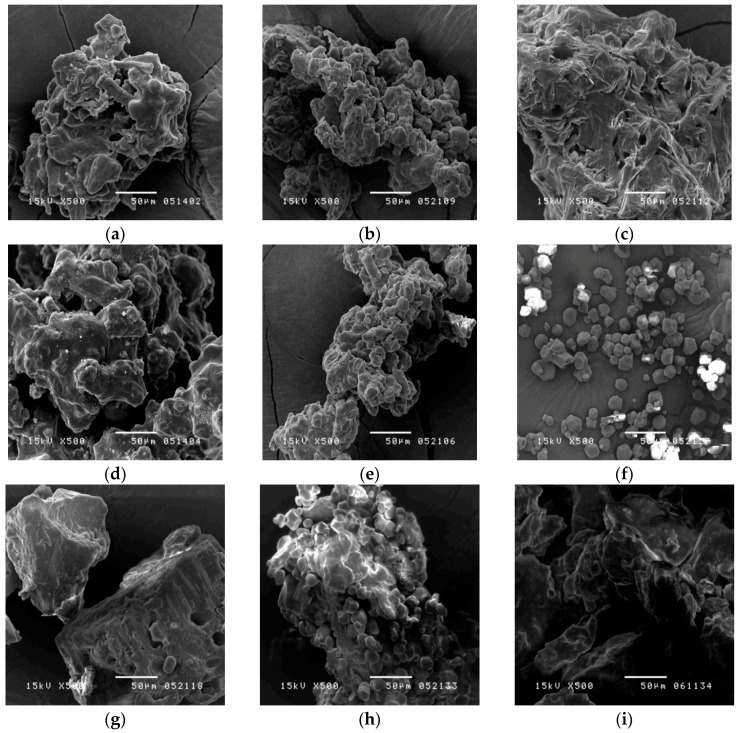
Scanning electron microscopy photographs of Sheng-Mai-San (**a**–**e**) products of brands A–E, respectively; (**f**) cornstarch; (**g**) raw herbal powder; (**h**) mixture cornstarch/raw herbal powder = 1:1; (**i**) mixture raw herbal powder/brand A SMS product = 1:1. (magnification 500×).

To observe the morphology of the samples, a scanning electron microscope (SEM) was used. Figure 3a–e show the outer appearance of the herbal pharmaceutical products, which feature a rough and irregular surface with sharp layers. In comparison with the SMS products, it was found that cornstarch was granular, uniform, and polygonal in shape (Figure 3f) [23], whereas the raw herbal powder was rough, irregular, and lumpy (Figure 3g). By SEM observation of the morphologies, the SMS products, starch, and raw herbal powder could be distinguished clearly. However, it is difficult to identify whether the raw herbal powder was added into the SMS products.

**Figure 4:**
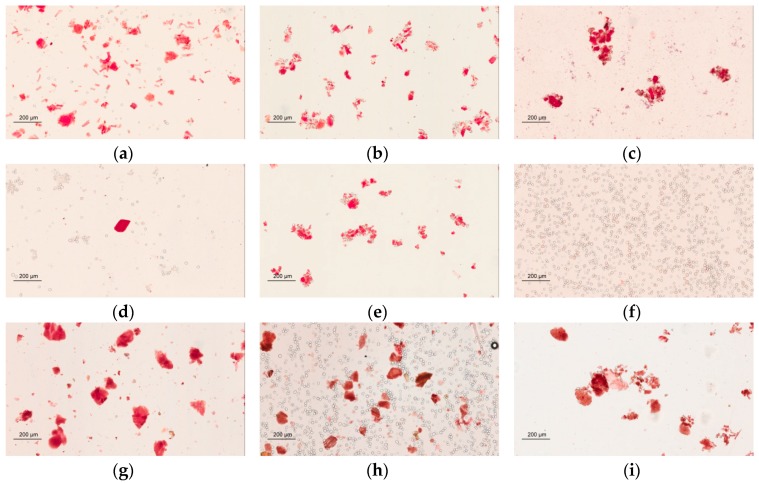
Microscopy photographs with Congo red staining. Light microscope photographs of Sheng-Mai-San (**a**–**e**) products of brands A–E, respectively; (**f**) cornstarch; (**g**) raw herbal powder; (**h**) mixture cornstarch/raw herbal powder = 1:1; (**i**) mixture raw herbal powder/brand A SMS herbal pharmaceutical product = 1:1 ( magnification 100×). Scale bar is 200 μm.

Congo red has a strong interaction with polysaccharides by noncovalent affinity and synthesizes a red complex [24]. An Aperio ScanScope slide scanner was used for the observation and identification of the cellulose fibers via Congo red staining. The photographs show that Sheng-Mai-San products made from different manufacturers (Figure 4a–e) and raw herbal powder (Figure 4g) were red or pink but cornstarch was not (Figure 4f). The results indicate that samples A to E contain fiber components, suggesting the possible use of raw herbal powder or cellulose fiber as additives.

**Figure 5:**
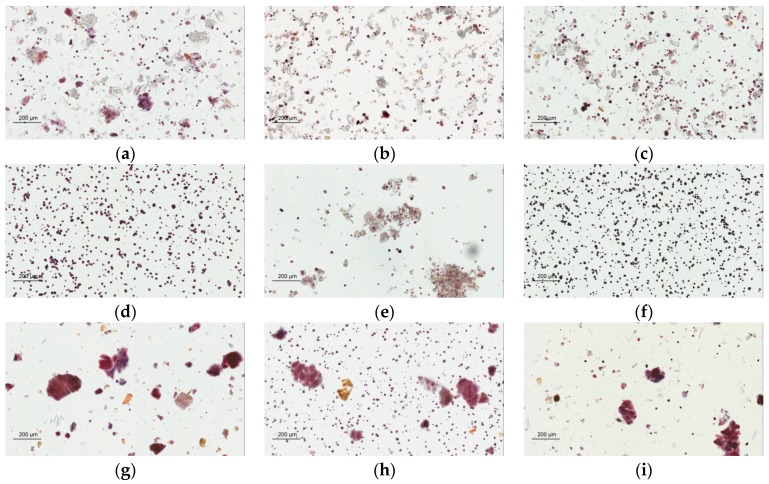
Microscopy photographs with iodine solution staining. Light microscopy photographs of (**a**–**e**) SMS herbal pharmaceutical products of brands A–E, respectively; (**f**) cornstarch; (**g**) raw herbal powder; (**h**) mixtrure cornstarch/raw herbal powder = 1:1; (**i**) mixture raw herbal powder/brand A SMS herbal pharmaceutical product = 1:1 (magnification 100×). Scale bar is 200 μm.

Iodine-KI solution is used in a common test for starch. In the presence of iodine-KI, amylose in starch is responsible for the formation of a deep blue color. The triiodide ion complex slips inside the coil of starch, creating an intense blue-black color. The starch identification method was assessed by light microscopy photographs using iodine-KI reagent staining in this study. The photographs show that the SMS products made by different manufacturers (Figure 5a–e) and cornstarch (Figure 5f) were blue or violet. It was found that the starch contents of samples A–E could be clearly observed under the microscope using iodine-KI reagent staining, and Aperio ImageScope software could be used to calculate the number of particles of cornstarch (100–400 mm^2^) or large starches from herbs (>400 mm^2^) within a fixed area. The result indicates that brands A to E contain cornstarch in particle numbers of 115–1267 and particle sizes of 173.1–206.7 mm^2^ (Table 5). Specifically, comparing brand D and pure cornstarch, the number of cornstarch particles were similar, indicating that brand D has a high cornstarch content. The cornstarch content in the brand D powder was calculated as approximately 79%. The results also reveal that brand A contains not only cornstarch but also herbal starch. The raw herbal powder contains large starches and no cornstarch; the particles were 2535.9 ± 270.3 mm^2^ in size, corresponding to herbal starch but being much larger than cornstarch.

Swelling, or an increase in volume, is observed as starch absorbs water. Determining the volume change of starch granules indicates the degree of hygroscopic swelling. Differences among starch granules may also result in different patterns of swelling power and solubility [23]. Generally, cornstarch is added into commercial herbal powders as an excipient to increase the stability and dispersity. The results demonstrate that the solubility of herbal pharmaceutical powder produced by manufacturers A–E was 0.91%–6.66% at 55 °C, 2.67%–6.50% at 65 °C, 3.04%–6.27% at 75 °C, 3.35%–6.16% at 85 °C, and 3.06%–12.27% at 95 °C (Table 6). Importantly, the SP of sample D was 0.91 to 12.27%, indicating that the SP increased with temperature. This finding is in agreement with the literature, which has reported that the SP of cornstarch is proportional to temperature [25]. Solubility testing was used to evaluate the water solubility at different temperatures among the herbal samples made by different manufacturers. The results demonstrate that the solubility of herbal pharmaceutical powder produced by manufacturers A–E was 39.93%–68.02% at 55 °C, 43.07%–69.39% at 65 °C, 39.92%–65.14% at 75 °C, 45.91%–66.65% at 85 °C, and 47.23%–71.02% at 95 °C (Table 7). Theoretically, the amylose and amylopectin molecules swell or dissolve gradually, and the water solubility of starch may increase with increasing temperature [26,27]. Therefore, the WSI will increase with the relative proportion of starch content. In samples A–C and E, no significant increase in the solubility was observed with increasing temperature. However, the WSI of sample D increased with temperature. These results suggest that sample D contains abundant starch.

The addition of raw herbal powder was confirmed by crude fiber analysis [24]. The crude fiber content (%) of samples A, B, C, D, and E was 19.81% ± 0.61%, 19.60% ± 0.26%, 12.40% ± 0.18%, 0.26% ± 0.04%, and 19.23% ± 1.20%, respectively (Table 8). Meanwhile, the crude fiber contents of the raw herbal powder and cornstarch/raw herbal powder (1:1) were 21.12% ± 0.61% and 9.52% ± 0.98%, respectively. Because raw herbal powder is mainly obtained from herbal plants, there is more cellulose fiber in the raw herbal powder than in the preparations. Excluding sample D, the crude fiber contents for the SMS products (A–C and E) were between the values for the raw herbal powder mixed with cornstarch (1:1) and the pure raw herbal powder, suggesting that these products all contain different proportions of raw herbal powder. In the literature, cornstarch was reported to contain almost no crude fiber [23]. Therefore, sample D contained less than 50% raw herbal powder. According to the previous study, crude fiber analysis was considered as a creditable indicator for the determination of the raw herbal powder added to a compound [24].

## 3. Materials and Methods

### 3.1. Chemical and Reagents

Ginsenosides Rb_1_, Rb_2_, Rc, Rd, Re, Rg_1_, Rh_1_ and compound K were purchased from ChromaDex (Irvine, CA, USA). Ophiopogonin D was purchased from TAUTO (Shanghai Tauto Biotech Co. Ltd., Shanghai, China). Schizandrin, 5-methoxyflavone and digoxin were obtained from Sigma-Aldrich Chemicals (St. Louis, MO, USA). LC/MS grade acetonitrile as obtained from J. T. Baker. Inc. (Phillipsburg, NJ, USA). Triple-deionized water (Millipore, Bedford, MA, USA) was used in the study. The root parts of *Panax ginseng* and *Radix ophiopogonis*, and the fruit parts of *Fructus schisandrae* were purchased from Zheng-Yuan herbal and traditional Chinese medicine store, Taipei, Taiwan. After comparing with the specimens in the National Research Institute of Chinese Medicine of Taiwan, the purchased herbs were the same as the specimens. Five different commercial pharmaceutical products of Sheng-Mai-San were included: SunTen Pharmaceutical Co., Ltd. (Taipei, Taiwan), Kaiser Pharmaceutical Co., Ltd. (Tainan, Taiwan), Chuang-Song-Zong Pharmaceutical Co., Ltd. (Kaohsiung, Taiwan), ShengFoong Co., Ltd. (Taipei, Taiwan), and ShengChang Pharmaceutical Co., Ltd. (Taipei, Taiwan). When presenting the analysis results, numeral codes were used to represent the names of these manufacturers. This study didn't be funded by any of the pharmaceutical herbal products of these manufacturers.

### 3.2. Analysis Condition

An UHPLC-MS/MS instrument equipped with an electrospray ionization interface (Waters Xevo TQ MS, Milford, MA, USA) was coupled to a UHPLC system (Waters Acquity BSM). The optimized MS conditions are shown in Table 9. MS/MS was conducted in selected reaction monitoring (SRM) mode. The chromatographic separation was achieved by using a C18 column (2.1 mm × 100 mm, particle size 1.7 μm; Waters Acquity UHPLC BEH). The column temperature was maintained at 40 °C. The mobile phase consisted of water (solvent A) and acetonitrile (solvent B), and the gradient program was shown as follows: 0–13 min: 5%–95% B; 13–14 min: 95%–5% B; 14–15 min: 5%–5% B *v*/*v*. The flow rate was 0.2 mL/min, and the injection volume was 10 μL.

### 3.3. Preparation of Standard Solution

The standard stock solutions for ginsenosides Rb_1_, Rb_2_, Rc, Rd, Re, Rg_1_, Rh_1_, compound K, ophiopogonin D, schizandrin, 5-methoxyflavone (internal stand 1), and digoxin (internal standard 2) were prepared as 1 mg/mL in methanol. All stock solutions were stored at −20 °C before use. The stock solutions were appropriately diluted to prepare a series of standard working solutions. 5-Methoxyflavone and digoxin were used as internal standards in ESI positive and negative modes, respectively. The internal standard concentrations of 5-methoxyflavone (0.2 μg/mL) and digoxin (2 μg/mL) were used as the working solution. Working solutions for the quality control samples at three concentrations were prepared in the same manner. All the solutions were stored at 4 °C and then brought to room temperature before analysis.

### 3.4. Sample Preparation for Extracts of Herbal Pharmaceutical Powders

*Panax ginseng* (30.0 g), *Radix ophiopogonis* (30.0 g) and *Fructus schisandrae* (15.0 g) were mixed together and extracted with 700 mL of boiling water for 30 min. The decoction was filtered, and the solution was evaporated under vacuum. Each sample was prepared with 0.1 g the commerical pharmaceutical SMS products or dried Sheng-Mai-San decoction powder immersed in 25 mL of methanol (4 mg/mL), extracted for 15 min in ultrasonic vibration machine, and centrifuged at 13,000 rpm for 10 min at 4 °C. After the filtrate was passed through a 0.22 μm syringe filter, the diluted filtrate was determined by UHPLC-MS/MS for analysis.

### 3.5. Quantitative Determination of Marker Compounds

The most intense ion detection was selected for quantitative determination. The relative concentrations of the marker compounds in each sample were calculated by interpolation from the calibration curve, and each batch of samples was in compliance with the same calibration curve. Using back calculation, the content of marker compounds in Sheng-Mai-San was calculated using the following formula: the content of biomarker compounds in Sheng-Mai-San (mg/g) = [observed concentration (ng/mL)/concentration of sample (4 mg/mL)] × dilution ratio.

### 3.6. Validation of Analytical Method

The validation of this method was based on the FDA Bioanalytical Method Validation publication [28]. The five-point calibration curves were based on the ratio of the peak area of the analytes and the internal standard. The correlation coefficients (*r*^2^) of all calibration curves were linearity and the values were greater than 0.995. The limit of quantification (LOQ) was determined by diluting standard samples of different concentrations, denoted as LOQ samples. The definition of the limits of detection was the signal to noise ratio (S/N) was required at least 3. The assessments of the precision and accuracy of the inter-day and intra-day were using the percentage of relative standard deviation and bias. The determination of the intra-day and inter-day variation were observed by analyzing six replicates on the same day and one consecutive days, respectively. The formula for calculating the percentage of relative standard deviation was the RSD (%) = (standard deviation (SD)/C_obs_) × 100. The formula of the percentage of bias was (%, bias) = [(C_obs_ − C_nom_)/C_nom_] × 100. The meaning of symbols of C_obs_ and C_nom_ were the mean value of the observed concentrations and the nominal concentration. Acceptable values of the accuracy and precision were less than ±15%, and ±20% at LOQ for all analytes.

### 3.7. Physical Examination of Additives in Raw Herbal Powder

#### 3.7.1. Scanning Electron Microscopy

Scanning electron microscopy (JEOL JSM-5300, Jeol Ltd., Tokyo, Japan) was used for observing the morphology of the powders of SMS products [18]. The herbs of Sheng-Mai-San herbs were purchased from a traditional herbal store in Taipei. The crushed herbs and cornstarch (Sun Right Co., Ltd., Nantou, Taiwan) powders were filtered through a 60 mesh sieve. For sample preparation, the SMS powder was dried at 40 °C for 24 h. The powders were set on an aluminum holder with glue, and then gold powder was coated on the samples by a gold sputter module for 90 s in a high vacuum evaporator (JFC-1200 Ion Sputterer, Jeol Ltd., Tokyo, Japan). Finally, the coated samples were analyzed under a scanning electron microscope.

#### 3.7.2. Light Microscopy Photographs of Congo Red and Iodine-KI Stained Sample

Light microscopy images were acquired by an Aperio ScanScope CS scanner (Aperio, Singapore). Samples were suspended in the mixed solution (glycerol/20% ethanol (1:1)), and placed on the microslides. The microslide covered with a coverslip that had been cleared of bubbles, and stained with 0.1% Congo red or 2% iodine-KI solution. For the Congo red stained slides, the red or pink staining was viewed under a 100× microscope magnification. For the iodine solution stained slides, blue and purple staining was viewed under a 100× microscope magnification.

#### 3.7.3. Swelling Power and Water Solubility Index

The swelling power and water solubility index of the herbal pharmaceutical powders were determined using a modification of the method of Roach, and Hoseney and Lu et al. [24,29,30]. The SMS powder sample (0.45 g) was suspended in 30 mL of distilled water. The SMS sample solution was vortexed and heated to 55, 65, 75, 85, and 95 °C sequentially for one hour each by a circulating water bath (BH-230D, YIN DER Instruments Co., Ltd., New Taipei, Taiwan)The heated samples were cooled to room temperature in an ice bath and then centrifuged at 8000× *g* for 20 min. To dry the supernatants, a heating oven was used. The weights of the residue and the SMS powder weight were denoted W1 and W2, respectively. The residue precipitate in the centrifuge tube was denoted as W3. SP and WSI were computed using the following formulas: WSI = (W_1_/W_2_) × 100%; SP = (W_3_ − W_2_)/W_2_ (1 − WSI/100).

#### 3.7.4. Crude Fiber Analysis

The crude fiber content was determined by the Crude Fiber in Flours, Feeds, and Feedstuffs Method No. 32-10.01 of the 11th edition of the American Association of Cereal Chemists (AACC), available online [31]. The SMS powder sample (2 g) was boiled in 200 mL of 1.25% H_2_SO_4_ for 30 min, then washed with hot distilled water. The sample solution was filtered with a suction apparatus. The residue was transferred to boiling 1.25% NaOH solution and treated in the same manner. This method was modified according to our previous study [24]. After cooling to room temperature, the residue was desiccated at 100 °C for 24 h to constant weight. The desiccated residue was placed in a muffle furnace (DF202, DENGYNG Instruments Co., Ltd, New Taipei City, Taiwan) at 550 °C–600 °C for 5–6 h until grey ash was obtained. The crude fiber (%) was calculated as the following formula: crude fiber (%) = (the constant weight of residue − the weight of ash) × 100/the weight of SMS sample.

#### 3.7.5. Statistical Analysis

Statistical analyses were performed using SigmaPlot software (version 13.0). The microscopy photograph analysis was performed with an Aperio ScanScope CS (Aperio Technologies, Vista, CA, USA), and the software of Aperio ImageScope (version 10.0) used for calculating the number of interesting particles. Data are presented as the mean ± standard deviation. Analysis of variance followed by Student’s *t* test or one-way ANOVA comparison adjustment was used, and statistically significant differences were defined as *p* < 0.05.

## 4. Conclusions

This study develops chemical and physical methods for evaluating the quality of pharmaceutical herbal products, Sheng-Mai-San. A sensitive, rapid and selective UHPLC/MS/MS method was developed and validated for the simultaneous determination of ginsenoside Rb_1_, Rb_2_, Rc, Re, Rd, Rg_1_, Rh_1_, compound K, ophiopogonin D and schizandrin in commercially available Sheng-Mai-San. Our study is the first report of the quantification of cornstarch using Aperio ImageScope software to calculate the particle number and size of the starch. This method can be applied for the quality control of commercial herbal powder products. The method developed in this study can provide a standard procedure for the quantity control of Chinese Sheng-Mai-San herbal pharmaceutical products.

## Figures and Tables

**Table 1:** LC-MS/MS results for the identification of the ten constituents.

Constituents	RT ^a^ (min)	Mass Fragments		Cone Voltage (eV)	Collision Energy (eV)
		Precursor Ion (amu)	Product Ion (amu)		
Ginsenoside Rb_1_	3.79	1131.78 [M + Na]^+^	789.41	100	56
Ginsenoside Rb_2_	4.05	1101.78 [M + Na]^+^	789.40	94	54
Ginsenoside Rc	3.92	1101.78 [M + Na]^+^	789.46	88	58
Ginsenoside Rd	4.41	969.65 [M + Na]^+^	789.40	94	54
Ginsenoside Re	2.89	969.65 [M + Na]^+^	789.41	94	44
Ginsenoside Rg_1_	2.91	823.63 [M + Na]^+^	643.34	84	36
Ginsenoside Rh_1_	4.35	637.48 [M − H]^−^	475.32	52	24
Ginsenoside Compound K	7.52	645.54 [M + Na]^+^	465.32	74	30
Ophiopogonin D	6.92	877.61 [M + Na]^+^	447.20	100	50
Schizandrin	5.85	433.29 [M + H]^+^	384.21	24	20

^a^ RT, retention time.

**Table 2:** Linear ranges, calibration curves, correlation coefficients (*r*^2^), and quantification and detection limits of ten constituents using liquid chromatography tandem mass spectrometry (LC-MS/MS).

Constituents	Calibration Curves	*r*^2^	LOQ (ng/mL)	LOD (ng/mL)
Ginsenoside Rb_1_	*y* = 0.0059*x* − 0.047	0.999	25	10
Ginsenoside Rb_2_	*y* = 0.0156*x* − 0.6078	0.998	50	10
Ginsenoside Rc	*y* = 0.0235*x* − 0.9627	0.998	50	10
Ginsenoside Rd	*y* = 0.0108*x* − 0.0355	0.999	10	5
Ginsenoside Re	*y* = 0.011*x* − 0.0143	0.998	25	10
Ginsenoside Rg_1_	*y* = 0.0018*x* − 0.0121	0.999	10	5
Ginsenoside Rh_1_	*y* = 0.002*x* − 0.0305	0.999	50	10
Ginsenoside Compound K	*y* = 0.0285*x* + 0.0203	0.999	25	10
Ophiopogonin D	*y* = 0.0309*x* + 0.0775	0.998	10	2.5
Schizandrin	*y* = 0.0126*x* − 0.2304	1.000	50	10

**Table 3:** Intra-day and inter-day precision and accuracy for the determination of ten constituents from standard samples.

Nominal Concentration (ng/mL)	Inter-day			Intra-day		
	Observed Concentration (ng/mL)	Precision (%)	Accuracy (%)	Observed Concentration (ng/mL)	Precision (%)	Accuracy (%)
Ginsenoside Rb_1_						
50	53.10 ± 4.20	6.21	7.90	49.50 ± 1.76	3.56	−0.99
100	93.23 ± 2.98	3.20	−6.77	99.76 ± 3.04	3.04	−0.24
500	498.8 ± 2.8	0.56	−0.24	499.8 ± 1.1	0.21	−0.03
Ginsenoside Rb_2_						
50	55.50 ± 3.29	5.92	11.01	50.13 ± 4.20	8.37	0.27
100	92.77 ± 5.10	5.49	−7.22	101.6 ± 5.7	5.58	1.61
500	498.6 ± 1.6	0.31	−0.28	505.5 ± 9.9	1.97	1.10
Ginsenoside Rc						
50	53.51 ± 1.32	2.47	7.03	51.17 ± 1.03	2.00	2.33
100	93.73 ± 2.62	2.79	−6.27	97.39 ± 5.12	5.26	−2.61
500	499.1 ± 2.7	0.54	−0.19	500.4 ± 5.3	1.07	0.08
Ginsenoside Rd						
50	54.54 ± 1.77	3.25	9.08	50.77 ± 3.62	7.14	1.54
100	98.14 ± 3.13	3.19	−1.85	100.3 ± 4.1	4.08	0.26
500	507.1 ± 13.8	2.73	1.41	502.9 ± 7.9	1.57	0.58
Ginsenoside Re						
50	51.09 ± 2.90	5.67	2.19	50.47 ± 2.61	5.18	0.95
100	98.92 ± 4.49	4.53	−1.49	99.80 ± 3.45	3.45	0.50
250	252.4 ± 10.0	3.97	0.94	252.1 ± 5.9	2.32	0.83
Ginsenoside Rg_1_						
10	10.63 ± 1.33	12.06	0.26	9.65 ± 0.95	9.85	−3.46
50	48.60 ± 2.06	4.25	−2.80	50.08 ± 2.33	4.66	0.15
100	100.8 ± 1.0	1.03	0.81	98.99 ± 2.74	2.76	−1.01
Ginsenoside Rh_1_						
50	49.26 ± 8.02	8.02	3.56	49.65 ± 1.93	3.88	−0.69
100	100.9 ± 6.6	6.50	0.90	102.9 ± 3.1	2.98	2.94
500	492.6 ± 12.3	2.49	−1.48	499.5 ± 9.9	1.99	−0.13
Ginsenoside Compound K						
25	25.17 ± 1.56	6.21	0.69	25.85 ± 0.82	3.18	3.41
100	100.4 ± 1.77	1.77	0.34	98.69 ± 1.23	1.25	−1.31
250	249.8 ± 0.5	0.21	−0.07	250.4 ± 0.4	0.17	0.15
Ophiopogonin D						
10	10.21 ± 0.55	5.34	2.12	9.56 ± 0.75	7.85	−4.44
50	49.69 ± 0.99	2.00	−0.61	51.03 ± 1.42	2.79	2.07
100	100.4 ± 0.7	0.71	0.44	99.97 ± 0.70	0.70	−0.03
Schizandrin						
50	56.34 ± 0.85	1.51	12.71	55.43 ± 0.25	0.45	10.86
100	92.81 ± 1.03	1.11	−7.19	93.01 ± 1.81	1.95	−6.99
500	500.7 ± 0.46	0.09	0.13	499.4 ± 2.2	0.45	−0.13

Note: data are expressed as the mean ± standard deviation (*n* = 6); Precision: relative standard deviation (RSD, %) = [standard deviation/C_obs_] × 100; Accuracy: Bias (%) = [(C_obs_ − C_nom_)/C_nom_] × 100.

**Table 4:** Contents of ten constituents in different brands of Sheng-Mai-San products.

Constituents (μg/g)	S	A	B	C	D	E
Ginsenoside Rb_1_	9.44 ± 0.74	346.5 ± 6.2	6.10 ± 0.23	250.1 ± 4.93	ND	ND
Ginsenoside Rb_2_	6.57 ± 0.35	277.6 ± 10.1	41.52 ± 2.23	22.15 ± 1.02	ND	ND
Ginsenoside Rc	6.53 ± 0.50	544. 8 ± 16.6	56.67 ± 4.73	18.75 ± 1.14	ND	ND
Ginsenoside Rd	ND	244.8 ± 8.6	19.58 ± 0.40	86.45 ± 5.40	ND	ND
Ginsenoside Re	ND	738.7 ± 6.8	26.46 ± 2.44	115.9 ± 7.29	ND	ND
Ginsenoside Rg_1_	4.60 ± 1.67	114.6 ± 5.4	15.26 ± 3.07	13.2 ± 1.67	ND	ND
Ginsenoside Rh_1_	550.6 ± 7.6	118.4 ± 4.0	52.78 ± 5.84	63.18 ± 2.59	33.38 ± 3.03	126.6 ± 15.2
Ginsenoside Compound K	ND	ND	ND	ND	ND	ND
Ophiopogonin D	ND	16.4 ± 1.1	ND	ND	4.34 ± 0.59	2.65 ± 0.45
Schizandrin	7.39 ± 0.29	722.5 ± 24.8	1532 ± 136	965.0 ± 44.0	409.8 ± 6.0	984.2 ± 30.1

The sample brands A–E represent the Sheng-Mai-San products were purchased from five different manufacturers; S represents the fresh sample prepard in the laboratory. ND, not detectable.

**Table 5:** Evaluation of iodine-KI stained slides of Sheng-Mai-San samples A–E, cornstarch and raw herbal powder by the Aperio ImageScope software.

Sample Brands	Particle Size > 400 mm^2^		Particle Size 100–400 mm^2^	
	Amount	Size (mm^2^)	Amount	Size (mm^2^)
A	42.3 ± 4.0	801.9 ± 34.7	174.3 ± 3.8	193.0 ± 8.3
B	-	-	166.3 ± 6.1	187.8 ± 3.7
C	-	-	362.0 ± 28.0	185.5 ± 1.3
D	-	-	1267 ± 20	173.1 ± 7.3
E	-	-	115.0 ± 4.9	206.7 ± 12.7
Cornstarch	-	-	1595 ± 41	183.0 ± 0.8
Raw herbal powder	34.7 ± 2.3	2536 ± 270	-	-

The sample brands A–E represent the Sheng-Mai-San products were purchased from five different manufacturers.

**Table 6:** Swelling power of Sheng-Mai-San samples A–E.

Sample Brands	Swelling (%)				
	55 °C	65 °C	75 °C	85 °C	95 °C
A	6.66 ± 0.26	6.50 ± 0.07	6.27 ± 0.59	6.16 ± 0.30	6.06 ± 0.82
B	3.27 ± 0.40	3.20 ± 0.29	3.99 ± 0.43	3.50 ± 0.29	3.06 ± 0.23
C	3.20 ± 0.88	3.11 ± 0.08	3.04 ± 0.52	3.35 ± 0.85	3.90 ± 0.34
D	0.91 ± 0.07	2.67 ± 0.18	6.79 ± 0.61	7.68 ± 1.62	12.27 ± 0.03
E	2.99 ± 0.22	4.19 ± 0.58	4.26 ± 0.24	3.75 ± 0.23	3.25 ± 0.19

The sample brands A–E represent the Sheng-Mai-San products were purchased from five different manufacturers.

**Table 7:** Water Solubility index of Sheng-Mai-San sample A–E.

Sample Brands	Solubility (%)				
	55 °C	65 °C	75 °C	85 °C	95 °C
A	65.04 ± 2.60	64.46 ± 2.21	63.63 ± 2.61	65.16 ± 2.59	71.02 ± 4.40
B	44.40 ± 4.47	43.07 ± 2.31	42.85 ± 4.41	45.91 ± 2.54	50.27 ±2.57
C	68.02 ± 2.54	69.39 ± 1.90	65.14 ± 2.55	66.59 ± 3.35	70.96 ±4.29
D	48.78 ± 4.38	48.39 ± 2.69	56.15 ± 2.58	60.62 ± 4.44	62.14 ± 0.14
E	39.93 ± 4.51	43.22 ± 1.93	39.92 ±0.07	47.38 ± 2.54	47.23 ± 2.57

The sample brands A–E represent the Sheng-Mai-San products were purchased from five different manufacturers.

**Table 8:** Crude fiber analysis of samples A–E and cornstarch/raw herbal powder (1:1) of SMS.

Sample Brands	S	A	B	C	D	E	Cornstarch:Raw Herbal Powder = 1:1
Crude fiber (%)	21.12 ± 0.61	19.81 ± 0.67	19.60 ± 0.26	12.40 ± 0.18	0.26 ± 0.04	19.23± 1.20	9.52 ± 0.98

The sample brands A–E represent the Sheng-Mai-San products were purchased from five different manufacturers; S represents the sample fresh prepare in the laboratory.

**Table 9:** LC-MS/MS analysis parameters.

Parameters	Value ESI(+)	Value ESI(−)
Source temperature (°C)	150	150
Desolvation temperature (°C)	400	400
Desolvation gas flow (L/h)	800	800
Cone gas flow (L/h)	50	50
Capillary voltage (kV)	3.60	2.64
Collision gas flow (mL/min)	0.15	0.15
Cone voltage (V)	60	30
Collision (V)	20	20
